# Dr. George Waldron

**Published:** 1907-10

**Authors:** 


Dr. George Waldron, of Rochester, N. Y., was instantly killed in an automobile accident August 28, 1907. He graduated at the Medical Department, University of Buffalo, in the class of 1881.

